# Transmission of Disease

**Published:** 1858-10

**Authors:** 


### THE EPITOME OF SANITARY LITERATURE.

Transmission of Diseases.*

Dr. Lauder Lindsay has published a valuable paper, full of rational suggestion and thought, on the Transmissions of Diseases between man and animals. We give, in the following, the summary of his arguments.

"1. That certain human diseases occur, under certain modifications—which are, however, often comparatively insignificant—in various of the lower animals, both wild and domesticated. Such diseases are cholera, yellow-fever, typhus and typhoid fevers, plague, small-pox, measles, croup, pleuropneumonia, influenza.

"2. That certain 'epidemic constitutions' of the atmosphere affect equally, or, at least, in marked degree, man and the lower animals; or, in other words, that a relation frequently subsists between coincident epidemics and epizootics, or epizootics preceding or following epidemics and such epidemics.

"3. That certain human diseases are transmissible, by contagion or otherwise, to various of the lower animals.

"4. That certain diseases of the lower animals are transmissible to different individuals, species, and genera of these animals.

"5. That certain diseases of the lower animals are transmissible, by contagion or otherwise, to man.

---

* On the Transmission of Diseases between Man and the Lower Animals. By W. Lauder Lindsay, M.D.

"6. That certain diseases, communicated to man from the lower animals, are propagable from man to man in the same manner as ordinary contagious human diseases are.

"7. That there are certain modifications in type, character, and degree in transmitted diseases, depending on the habits and structure of the animals affected; and that such modifications must be carefully studied, in order to a correct appreciation of comparative pathology.

"As subsidiary or collateral propositions, I would further state my conviction:—

"8. That the study of the diseases of the lower animals is calculated to throw much light on our knowledge of various obscure human diseases, and specially such as possess an epidemic character.

"9. That the same study has an important bearing on the health of our flocks and herds, the integrity of our agricultural interests at home and in the colonies, and on the use of the flesh of the domestic animals as food for man.

"10. That the pathology, especially, of the diseases of the lower animals, requires an extended and more accurate study in this country.

"11. That experimentation on the lower animals, as to the transmissibility of diseases to or from them and man, is one of the most promising means of arriving at sound conclusions as to the nature of various wide-spread diseases, both in them and in man.

"12. That, with a view to the proper conducting of experiments, it is necessary to make a selection of the animals to be operated upon, according to the nature of such experiments, or of the diseases to be studied."

